# Streamlining remote nanopore data access with *slow5curl*

**DOI:** 10.1093/gigascience/giae016

**Published:** 2024-04-12

**Authors:** Bonson Wong, James M Ferguson, Jessica Y Do, Hasindu Gamaarachchi, Ira W Deveson

**Affiliations:** Genomics and Inherited Disease Program, Garvan Institute of Medical Research, Sydney, NSW 2010, Australia; Centre for Population Genomics, Garvan Institute of Medical Research and Murdoch Children’s Research Institute,Sydney, NSW 2010, Australia; School of Computer Science and Engineering, University of New South Wales, Sydney, NSW 2052, Australia; Genomics and Inherited Disease Program, Garvan Institute of Medical Research, Sydney, NSW 2010, Australia; Centre for Population Genomics, Garvan Institute of Medical Research and Murdoch Children’s Research Institute,Sydney, NSW 2010, Australia; Genomics and Inherited Disease Program, Garvan Institute of Medical Research, Sydney, NSW 2010, Australia; Centre for Population Genomics, Garvan Institute of Medical Research and Murdoch Children’s Research Institute,Sydney, NSW 2010, Australia; School of Computer Science and Engineering, University of New South Wales, Sydney, NSW 2052, Australia; Genomics and Inherited Disease Program, Garvan Institute of Medical Research, Sydney, NSW 2010, Australia; Centre for Population Genomics, Garvan Institute of Medical Research and Murdoch Children’s Research Institute,Sydney, NSW 2010, Australia; School of Computer Science and Engineering, University of New South Wales, Sydney, NSW 2052, Australia; Genomics and Inherited Disease Program, Garvan Institute of Medical Research, Sydney, NSW 2010, Australia; Centre for Population Genomics, Garvan Institute of Medical Research and Murdoch Children’s Research Institute,Sydney, NSW 2010, Australia; St Vincent’s Clinical School, Faculty of Medicine, University of New South Wales, Sydney, NSW 2052, Australia

**Keywords:** nanopore, raw signal, signal data, cloud, sequencing data

## Abstract

**Background:**

As adoption of nanopore sequencing technology continues to advance, the need to maintain large volumes of raw current signal data for reanalysis with updated algorithms is a growing challenge. Here we introduce *slow5curl*, a software package designed to streamline nanopore data sharing, accessibility, and reanalysis.

**Results:**

*Slow5curl* allows a user to fetch a specified read or group of reads from a raw nanopore dataset stored on a remote server, such as a public data repository, without downloading the entire file. *Slow5curl* uses an index to quickly fetch specific reads from a large dataset in SLOW5/BLOW5 format and highly parallelized data access requests to maximize download speeds. Using all public nanopore data from the Human Pangenome Reference Consortium (>22 TB), we demonstrate how *slow5curl* can be used to quickly fetch and reanalyze raw signal reads corresponding to a set of target genes from each individual in large cohort dataset (*n* = 91), minimizing the time, egress costs, and local storage requirements for their reanalysis.

**Conclusions:**

We provide *slow5curl* as a free, open-source package that will reduce frictions in data sharing for the nanopore community: https://github.com/BonsonW/slow5curl.

## Background

Nanopore sequencing has become a key pillar in the genomic technology landscape. Platform updates from Oxford Nanopore Technologies (ONT) have enabled increasingly cost-effective sequencing of large eukaryotic genomes and transcriptomes [1, 2]. However, the nanopore community continues to be hampered by large data volumes and computational bottlenecks.

An ONT device measures the displacement of ionic current as a DNA or RNA molecule passes through a nanoscale protein pore. Time-series current signal data are recorded and “basecalled” into sequence reads or analyzed directly [1]. Algorithms for ONT basecalling and other signal-level analysis are continually evolving. For example, within a recent 1-year period, we observed a 0.5% decrease, or 8.8% relative improvement, in the mean error rate of an identical dataset basecalled with ONT’s *Guppy* v6.2.1 (July 2022) and v6.5.7 (May 2023; high-accuracy model; Supplementary Fig. S1). Rapid gains have also been made in the performance of DNA methylation detection (5mC and 5hmC), and many new tools for profiling diverse DNA and RNA modifications are released each year [3–8]. Therefore, to maximize the utility of a given dataset and to enable standardization over time, it is important to retain ONT raw signal data for future reanalysis. However, the raw data are large—roughly ∼1 TB for a typical human genome sample at ∼30× coverage (stored in POD5 or BLOW5 format), or ∼10× larger than the corresponding basecalled reads—which imposes significant costs during storage, retrieval, and reanalysis.

Cloud computing environments are increasingly popular platforms for genomics data storage and sharing. Many large, public ONT reference datasets (both existing and under construction) are hosted on the cloud, including the Human Pangenome Reference Consortium (HPRC) [9], Telomere-to-Telomere consortium [10], Singapore Nanopore Expression Project [11], 1000G ONT Sequencing Consortium, NIH Center for Alzheimer’s and Related Dementias [12], and Genome in a Bottle Consortium [13]. Open access to these resources is vital for the genomics community, but large file sizes can make access impractical for many users. Currently, a user wishing to reanalyze a gene/transcript/region(s) of interest within a reference sample must first download the entire >1 TB dataset to their local machine or their own cloud instance, necessitating large storage capacity, involving a high bandwidth connection, and incurring significant egress costs (usually borne by the host). These are significant frictions for reanalysis of even a single genome/transcriptome dataset and a major barrier for large cohort datasets.

To address this challenge, we have developed *slow5curl*, a simple command-line tool and underlying software library to improve remote access to nanopore signal datasets. *Slow5curl* enables a user to extract and download a specific read or set of reads (e.g., the reads corresponding to a gene of interest) from a dataset on a remote server, avoiding the need to download the entire file. *Slow5curl* uses highly parallelized data access requests to maximize speed. Here we show how *slow5curl* can facilitate targeted reanalysis of remote nanopore cohort data, effectively removing data access as a consideration.

## Results

### *Slow5curl* basic usage

**Figure 1:**
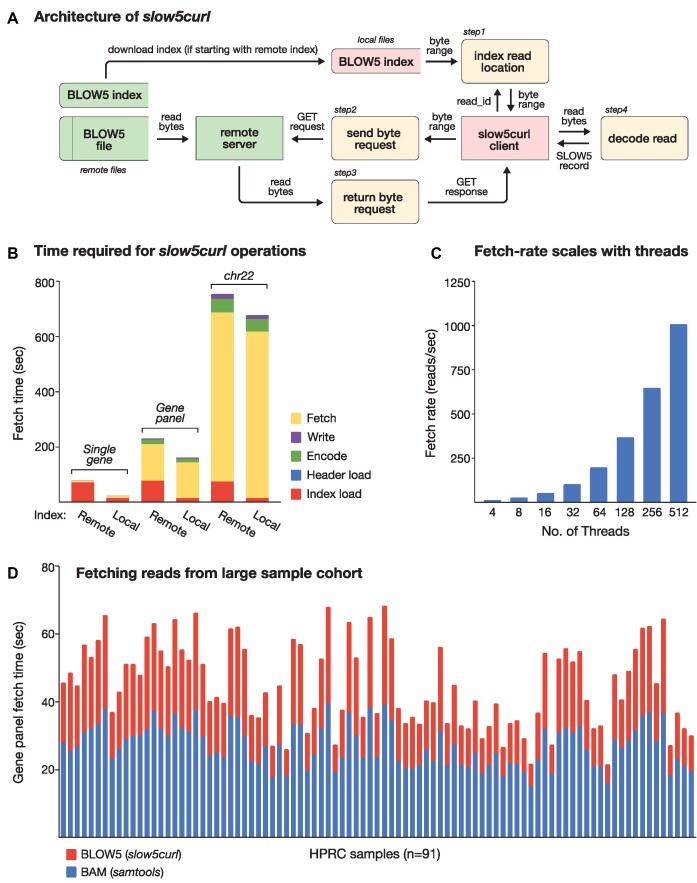
Evaluating remote nanopore data access performance with *slow5curl*. (A) Schematic summarizes the data flow between entities as *slow5curl* fetches a single read from a nanopore signal dataset on a remote server. The *slow5curl* client and the remote server are represented as separate entities. Locations of datasets (BLOW5, BLOW5 Index) are denoted by their respective entity colors (green = remote; red = local). The order of execution of processes (yellow) is indicated by their accompanying step numbers. (B) Time taken to fetch a group of signal reads from a remote whole-genome ONT sequencing file in BLOW5 format. Times are shown separately for 3 sets of reads, corresponding to a single gene (left), a hypothetical gene panel comprising 100 genes (center), and the entirety of chr22 (right). Times are shown separately for fetching reads using a remote versus local index, and overall times are broken down into the times taken for individual processes (“fetch,” “write,” “encode,” “header load,” “index load”). Values presented are an average of *n* = 10 independent measurements. (C) Rate with which reads are fetched from the same dataset (in reads/s) when invoking *slow5curl* with increasing numbers of threads (*n* = 4–512). (D) Time taken to fetch all signal reads corresponding to the hypothetical gene panel above from each of *n* = 91 whole-genome ONT sequencing datasets currently available via the HPRC. Times are shown separately for fetching basecalled alignments (BAM format; blue) and signal reads (BLOW5 format; red) with *samtools* and *slow5curl*, respectively.

The *slow5curl* (RRID: SCR_025117) command-line tool can fetch a specific read, or group of reads, from an ONT signal dataset in binary SLOW5 (BLOW5) format [14] stored on a remote server accessible by *http*/*https* or *ftp* protocols (Fig. 1A). BLOW5 is a compressed binary format with a simple file structure, which is suitable for streaming [14]. An accompanying index file describes the location of each read within the file, enabling efficient extraction of reads by random access pattern (Fig. 1A). The BLOW5 index may be stored remotely (either accompanying its BLOW5 file at the same URL or at another location specified by the user) or on the user’s local machine. The index is first downloaded (unless the user specifies a local index) and loaded into memory before querying the remote dataset (Fig. 1A). By default, the index will be downloaded to a temporary location and deleted by *slow5curl* after use. Alternatively, the user may retain it by specifying an option “*--cache*” and then provide it as a local index for subsequent commands. This avoids repeated downloading of the index when making multiple successive queries.

To fetch a single read or list of reads, based on their unique read IDs, the user may invoke *slow5curl get* as follows:

# get a single read with ID “05ef1592-a969-4eb8-b917-44ca536bec36”

$ slow5curl get https://url/to/reads.blow5 05ef1592-a969-4eb8-b917-44ca536bec36 -o fetched_read.blow5

# get a list of reads specified in file “readidlist.txt”

$ slow5curl get https://url/to/reads.blow5 --list readidlist.txt -o fetched_reads.blow5

In addition to *get*, the subtools *head* and *reads* may be used to print the header or a complete list of read IDs from a remote BLOW5 file, respectively.

### Fetching reads from a genomic region

A typical use case for *slow5curl* is to fetch the raw signal reads corresponding to a specific genomic region from a remote dataset. In doing so, the user may quickly reanalyze a gene/transcript of interest with the latest basecalling, DNA methylation profiling, or other signal-level analysis algorithms. Basecalled reads aligned to a reference genome/transcriptome (BAM format) must also be available, stored either locally or remotely, to provide genomic coordinates for a given read. Given their small size (12.7% compared to corresponding BLOW5 or 8.9% compared to FAST5 tarball), the additional cost to do so is relatively small (Supplementary Table S1). *Slow5curl* works similarly to the remote client feature in *samtools/htslib* [15], and the 2 tools may be used in tandem to retrieve raw signal reads for a specific region, as follows:

# get raw signal reads corresponding to genomic interval “chr1:1-1000000”

$ samtools view https://url/to/reads.bam chr1:1-1000000 | cut -f1 | sort -u > readidlist.txt

$ slow5curl get https://url/to/reads.blow5 --list readidlist.txt -o fetched_reads.blow5

To assess the performance of *slow5curl*, we measured the time taken to fetch all raw signal reads corresponding to a single gene (*BRCA1*), a hypothetical gene panel of 100 genes, or a complete chromosome (chr22) from a whole-genome ONT reference dataset hosted on our public AWS repository [16] (see Supplementary Table S1). Fetching reads for the single gene, gene panel, and complete chromosome took 88 seconds, 254 seconds, and 13 minutes, respectively, on a system with ∼3,000 Mbit/s Internet connection (Fig. 1B; see Supplementary Table S2). Roughly ∼70 seconds was required to download the remote BLOW5 index, constituting ∼95% of the total time for the single gene. However, this was reduced to ∼13 seconds when the index was cached locally (Fig. 1B). Notably, it took ∼3.2 hours to download the whole-genome dataset using the AWS Command Line Interface (AWS CLI): a significant unnecessary delay if intending to analyze only a subset. When repeated using different basecalling software versions (Guppy v6.5.7 vs. Dorado v7.2.13; HAC model), we observed high concordance in the list of reads mapped to each target region (single gene, 99.2%; gene panel, 99.3%; chr22, 98.4%), meaning the basecaller version has minimal impact on the group of reads retrieved by *slow5curl*.

### Efficient read-fetching by parallel threads

As shown previously [14], BLOW5 format permits efficient parallel file access by multiple CPU threads. *Slow5curl* also uses parallel access by multiple threads to maximize performance. However, this differs from the paradigm for processor-intensive applications, wherein the ideal number of threads is close to the number of physical CPU threads available. Instead, when fetching batches of reads over the network, it is ideal to invoke an excessive number of parallel requests (e.g., hundreds) in order to hide the latency of a given request (see Methods and Implementation).

To evaluate the multithreading strategy used in *slow5curl*, we repeatedly fetched all chr22 reads from the ONT dataset above, each time invoking an increasing number of threads (Fig. 1C). The rate of read-fetching scaled linearly with the number of threads used and did not reach a ceiling, even with 512 threads (which was the maximum connections allowed by the server; Fig. 1C). This is indicative of highly efficient parallelization, reducing the total time for extracting chr22 to just 294.74 seconds, of which 0.04% was loading the index (Supplementary Fig. S1A, B).

### Fetching reads from a large cohort

A key motivation for developing *slow5curl* was to enable efficient access to large, public reference datasets, such as HPRC [9]. HPRC’s data are currently stored in a publicly accessible AWS bucket. Raw ONT data are stored in FAST5 format with 1 large tarball for each individual dataset (Supplementary Table S3). FAST5 tarballs do not permit indexing or random access, meaning a user must download the entire dataset for a given individual in order to access reads for even just a single gene.

To demonstrate how *slow5curl* can address this issue, we first downloaded all ONT datasets currently available from HPRC (*n* = 91), converted them to BLOW5 format with indexes (reducing the average size by 29.7%), and then uploaded to commercial cloud storage (Wasabi cloud), along with accompanying basecalled alignments (see Supplementary Methods). From here, we used *samtools* and *slow5curl get* (as above) to remotely fetch all alignments and signal reads corresponding to our hypothetical gene panel, from each HPRC dataset (invoking *n* = 128 threads). We recorded both the time taken to fetch the reads of interest from each dataset and to re-basecall them with the latest *Guppy* version (via the *Buttery-eel* SLOW5 wrapper [17]; see Supplementary Table S3).

Fetching the specified reads (mean *n* = 3,308 reads) from each remote file took a mean of 45 seconds, and a total of ∼1.2 hours was required to traverse the entire cohort (Fig. 1D). The time required for each dataset scaled linearly with their total sizes (i.e., sequencing depth), meaning the fetching rate was stably maintained across the cohort (Supplementary Fig. S2A, B). Notably, the time required to basecall each set of extracted reads (mean 181 seconds) was significantly longer than its fetching time (Supplementary Fig. S2C; Supplementary Table S3). Since basecalling can be initiated on each individual set of reads without waiting for the subsequent set to be fetched, the overall time taken to complete this analysis is almost entirely determined by the basecalling time, and the net time added for data access with *slow5curl* becomes negligible. Similarly, the experiment would require downloading ∼22.5 TB of BLOW5 files to local storage, compared to ∼120.5 GB of reads fetched by *slow5curl*, dramatically reducing data egress costs incurred on most commercial cloud platforms. Availability of such large local storage capacity is also unrealistic for most users. In summary, this experiment demonstrates how *slow5curl* can be used to dramatically reduce the overheads for data access during reanalysis of ONT cohort data.

## Discussion

Data accessibility is critically important to the genomics community and a prerequisite for open, reproducible science. With the breadth of nanopore sequencing adoption and the scale of nanopore datasets growing rapidly, there is a need for new and efficient methods for nanopore data sharing and public access. *Slow5curl* allows a user to quickly fetch specific reads (e.g., for a gene of interest) from a raw nanopore signal dataset on a remote server, without downloading the entire dataset. This saves time and egress costs, and it reduces the need for a high-bandwidth connection and large local storage. *Slow5curl* makes it feasible for even low-resource users to fetch and reanalyze nanopore signal data from large cohort datasets like HPRC and, in doing so, increases the value of such initiatives.

The large size and complex file structure of ONT native signal datasets poses a particular challenge for genomics data repositories, such as EBI’s European Nucleotide Archive (ENA; RRID:SCR_006515) or NCBI’s Sequence Read Archive (SRA; RRID:SCR_004891). ONT’s FAST5 format is currently supported by ENA and SRA. However, users must upload a single FAST5 tarball for a given dataset, which is typically >1 to 2 TB for a standard PromethION (RRID:SCR_017987) sequencing run. A user wishing to access the data must then download and extract the entire file. Given these barriers, many nanopore users neglect to provide the raw data for published studies to SRA or other repositories, preventing reanalysis with updated basecalling, methylation profiling, or other signal-based analysis methods [3–8]. *Slow5curl* provides an improved solution for data repositories, analogous to the familiar *htslib*/*samtools* and *fqidx*/*faidx curl* protocols, which facilitate access to remote BAM and FASTQ data, respectively [15]. We anticipate that streamlined accessibility would encourage more users to share raw nanopore datasets on permanent public repositories.

In fetching specific reads from a remote dataset with minimal delay, *slow5curl* has the potential to enable interactive analysis and exploration of large nanopore signal datasets. For example, one can envision an interactive browser for signal data exploration, analogous to existing genome browsers that work with sequence-level data. While there are several current tools for visualizing nanopore signal reads, such as our own recent package *Squigualiser* [18], these require the dataset(s) under inspection to be stored locally, which is problematic for large nanopore datasets. *Slow5curl* provides a mechanism for interactive exploration of remote data, with reads being rapidly fetched, processed, and plotted as the user navigates the hypothetical browser. We show here that a cached local index would reduce the latency on this process to a matter of seconds. Further speed-ups are likely possible by integrating more specialized protocols, such as the S3 API, into *slow5curl*, although this would necessitate trade-offs in compatibility. We chose to use the standard *curl* library for its compatibility with any *http*/*https* or *ftp* hosted storage.

*Slow5curl* is the latest feature in the SLOW5 data ecosystem, a community-centric project designed to improve the usability of nanopore signal data [19]. The initiative is inspired by the SAM/BAM alignment data format and its many associated utilities, such as the remote client feature in *samtools/htslib* [15], which *slow5curl* emulates for nanopore signal data. Efficient, remote data access by *slow5tools* is possible thanks to the simple SLOW5/BLOW5 file structure and accompanying index, following similar design principles to SAM/BAM. In contrast, complex file formats like ONT’s original FAST5 or new native POD5 format do not support efficient random access or indexing, thereby prohibiting efficient remote data access. The SLOW5 data format [14] is now accompanied by software libraries in C/C++, python, rust, and R for reading/writing SLOW5 files [20]; the *slow5tools* package for creating, converting, handling, and interacting with SLOW5/BLOW5 files [21]; the *Buttery-eel* wrapper for ONT basecalling and methylation calling software [17]; the *Squigulator* [22] and *Squigualiser* [18] packages for simulation and visualization of signal data; and a range of other open source tools [7, 23–27].

Despite the advantages of SLOW5/BLOW5, ONT are yet to adopt the file format for direct reading/writing on their instruments or software. Therefore, we are committed to maintaining SLOW5 as a stable, standardized, well-documented, and open alternative to ONT’s native data formats. We provide *slow5curl* as a free and open resource to improve data accessibility for the nanopore community [28].

## Methods and Implementation

### Architecture and implementation of *slow5curl* library (*slow5curllib*)

The underlying library *slow5curllib* is written in C; it uses the file format library *slow5lib* and the multiprotocol file transfer library *libcurl*. Minimizing dependencies is a central design principle of the SLOW5 ecosystem. We therefore chose to develop *slow5curllib* as a separate library, rather than incorporating it into *slow5lib* or *slow5tools*, to avoid adding *libcurl* as a new dependency to these core SLOW5 packages.

Every SLOW5/BLOW5 file can be represented with a (much smaller) corresponding index file that maps every read ID to its respective location in memory. Since most RESTful APIs allow for byte-range fetches, *slow5curllib* takes advantage of this index file to send read-specific file transfers.

The library implements a single fetch (*s5curl_get()*) through the interface of *libcurl*. Once the BLOW5 header and its index are downloaded, we supply a connection handle to *libcurl* containing all the necessary configurations required to generate a byte-range request to the remote server. The thread making the call then waits until this request is fulfilled. *Slow5curl*’s batch fetch uses this exact method internally on parallel threads.

Batch fetches are a high-level multithreaded option for getting lists of reads quickly (using *s5curl_get_batch()*). *Slow5curllib* does this by spawning worker threads (C/C++ POSIX) to fetch reads in parallel. This way, we can accelerate high-volume fetch operations on multithreaded systems.

In very rare instances, for network-related reasons, 1 or more fetches within a batch will fail. Instead of aborting the method (since the library does not expose each worker thread), *slow5curllib* provides the option to retry any particular fetch a certain amount of times before it fails (default 1). Since it is usually an external issue, we also provide a parameter to control the amount of time to wait before retrying (default 1 second). If a fetch fails twice in a row, it is likely that something has gone wrong with the server/connection, or the client is being denied further access.

### Architecture and implementation of *slow5curl* tool

*Slow5curl* provides the functionality of the library through a command-line interface. Each *slow5curl get* command simply invokes the library method *s5curl_get()* unless provided with a list, where it will instead invoke *s5curl_get_batch()*. Additionally, *slow5curl* is able to provide BLOW5 file meta-data to the user. The *slow5curl head* command prints out the header downloaded from the remote BLOW5, and *slow5curl reads* prints out all read IDs stored in the BLOW5 index.

By default, *slow5curl* will automatically delete any downloaded BLOW5 index unless a permanent file path is specified through the --*cache* option. This option is for if the user requires to fetch data from a remote BLOW5 more than once. Downloading the index takes a nonnegligible amount of time, so caching it to a local path will avoid repeated downloads. After the index is cached, the user can provide a local index path through the --*index* option.

### Benchmark experiments

#### Datasets

The HG002 (NA24385) reference dataset used for the benchmarking (Supplementary Table S1) was prepared using the ONT LSK114 ligation library kit and was sequenced on an ONT PromethION on an R10.4.1 flow cell to generate ∼30× genome coverage. Sheared DNA libraries (∼17 Kb) were used. The FAST5 files were live-converted using the *real-f2s* script and then merged into a single BLOW5 (*zlib+svb-zd* compression) file and indexed using *slow5tools* [19]. Basecalling was performed using *Buttery-eel* (through Guppy v6.4.2) under the high-accuracy model. Reads were mapped to the hg38 reference using *Minimap2* (v2.17), and a sorted BAM file (with index) was created using *samtools*. The data were uploaded to the *gtgseq* AWS S3 bucket in the *US West (Oregon) us-west-2* region using AWS CLI.

The HPRC data (*n* = 91 samples) were downloaded from the human-pangenomics AWS S3 bucket. For each sample, the downloaded tarball of FAST5 files was extracted and then converted into a merged BLOW5 file (*zlib*+*svb-zd* compression) and indexed using *slow5tools*. The 31.2 TB of FAST5 tarballs reduced to 21.93 TB after the BLOW5 conversion (see Supplementary Table S3). The available basecalled data for each sample were also downloaded (FASTQ.gz format) from the human-pangenomics AWS S3 bucket and mapped to the hg38 genome using *Minimap2* (v2.17), then sorted and indexed using *samtools*. The BLOW5 files (with index) and BAM files (with index) for all the 91 samples were uploaded to an s3 bucket in the Wasabi cloud under the *Asia Pacific (Sydney) ap-southeast-2 region* using AWS CLI.

#### System information

A Dell PowerEdge C4140 server computer with a 10 Gb ethernet network connection was used for the experiments (Supplementary Table S2). The server is located in Sydney and was measured to have ∼3 Gbit/s download speed when benchmarked via *speedtest* by *ookla*.

#### Methodology for HG002 experiments

The HG002 dataset is hosted on the AWS S3 bucket in the *US West (Oregon) us-west-2* region and represents a high-latency scenario when being accessed from a computer located in Sydney.

We tested the performance impact of the number of reads fetched by *slow5curl* by providing read IDs corresponding to the region of the*BRCA1* gene (chr17:43044295-43170245), a hypothetical gene panel comprising 100 randomly selected genes, and *chr22* (the smallest human autosome). Each test was run on 128 threads, with the average time recorded from 10 runs. All runs were performed during low-network load conditions (on weekends).

#### Methodology for HPRC cohort experiments

This dataset is stored on the *Asia Pacific (Sydney) ap-southeast-2* region and represents a low-latency scenario when being accessed from a computer located in Sydney.

We test *slow5curl* on 91 samples alongside *samtools* to fetch all reads corresponding to a hypothetical gene panel comprising 100 randomly selected genes. This involves first using *samtools* to fetch the read IDs corresponding to the gene panel regions (BED format) into a read ID list. After this, we use *slow5curl* to fetch the reads into a BLOW5 file. Lastly, we basecall the reads using *Buttery-eel* (through *Guppy* v6.4.2) with the super-accuracy (SUP) model. This experiment was run during low network load conditions.

## Source Code Availability

*Slow5curl* is free and open source and can be accessed at [28]. The GitHub commit used for the benchmarks is 6d930a3a6cc3e206fbfc21c402a8fc59717cacfc.

Project name: *slow5curl*
Project homepage: https://github.com/BonsonW/slow5curl
Operating system(s): Linux, MacOS
Programming language: C
Other requirements: zlib
License: MIT
RRID: SCR_025117
Bio.tools: biotools:slow5curl

## Supplementary Material

giae016_GIGA-D-23-00403_Original_Submission

giae016_GIGA-D-23-00403_Revision_1

giae016_GIGA-D-23-00403_Revision_2

giae016_Response_to_Reviewer_Comments_Original_Submission

giae016_Response_to_Reviewer_Comments_Revision_1

giae016_Reviewer_1_Report_Original_Submission Jan Voges -- 2/12/2024 Reviewed

giae016_Reviewer_2_Report_Original_Submission Yunfan Fan -- 2/17/2024 Reviewed

giae016_Reviewer_3_Report_Original_Submission Guillermo Dufort y Alvarez -- 2/21/2024 Reviewed

giae016_Reviewer_3_Report_Revision_1 Guillermo Dufort y Alvarez -- 3/4/2024 Reviewed

giae016_Supplemental_Files

## Data Availability

The HG002 dataset in BLOW5 format used for benchmarking is available as part of the AWS Open Data Program in the *gtgseq* S3 bucket [29]. This dataset is also available under the NCBI SRA at Bioproject PRJNA744329. HPRC data are available in FAST5 format under the human-pangenomics AWS S3 bucket [30] and can be converted to BLOW5 format by following instructions in the Supplementary Methods section. Snapshots of our code and other data further supporting this work are openly available in the *GigaScience* repository, GigaDB [31].

## Additional Files

**Supplementary Fig. S1**. Assessing improvements in ONT basecalling software between *Guppy* versions. Frequency distributions show preread accuracy, as assessed by read/reference sequence identity on a Phred quality scale. Different distributions show accuracy achieved with different *Guppy* versions and fast versus HAC models all released within a ∼1-year period (*Guppy* v6.2.1 [July 2022] and v6.5.7 [May 2023]).

**Supplementary Fig. S2**. Evaluating the multithreading performance in *slow5curl*. (A) Time taken to fetch all reads corresponding to a hypothetical gene panel comprising 100 genes from a remote whole-genome ONT sequencing file in BLOW5 format, when invoking *slow5curl* with increasing numbers of threads (*n* = 4–512). Overall times are broken down into the times taken for individual processes (“fetch,” “write,” “encode,” “header load,” “index load”). (B) Same as above but times for each individual process are expressed as a fraction of the total runtime, in stacked bar format.

**Supplementary Fig. S3**. Evaluating *slow5curl* performance on large cohort datasets. (A) Time taken to fetch all signal reads corresponding to a hypothetical gene panel (*n* = 100 genes) from each of *n* = 91 whole-genome ONT sequencing datasets currently available via the HPRC, relative to the number of signal reads being extracted for each dataset, which varies depending on the sequencing depth for each HPRC sample. (B) Same as above but fetch times are shown relative to extracted file sizes (in MBytes). The linear correlation observed in these 2 plots indicates *slow5curl* maintained a stable rate of data read fetching across the full HPRC cohort. (C) Stacked bar chart shows the fraction of total time taken to fetch reads from each HPRC sample allocated to each individual component of the process (“fetch,” “write,” “encode,” “header load,” “index load”).

**Supplementary Table S1**. Data specifications.

**Supplementary Table S2**. Computer and connectivity specifications.

**Supplementary Table S3**. Statistics for the HPRC datasets.

## Abbreviations

AWS CLI: AWS Command Line Interface; ENA: European Nucleotide Archive; HPRC: Human Pangenome Reference Consortium; NCBI: National Center for Biotechnology Information; ONT: Oxford Nanopore Technologies; SRA: Sequence Read Archive.
