# Supplementary material for: Streamlining remote nanopore data access with slow5curl
Source: Gigascience. 2024 Apr 12;13:giae016. doi: 10.1093/gigascience/giae016 (PMC11010652; doi:10.1093/gigascience/giae016)
Supplement: giae016_Supplemental_Files [file giae016_supplemental_files.zip › Slow5curl_Supplement.pdf]

SUPPLEMENTARY MATERIAL

Streamlining remote nanopore data access with *slow5curl*

Bonson Wong, James M. Ferguson, Jessica Do, Hasindu Gamaarachchi*, Ira W. Deveson*

* Joint-senior authors; correspondence: hasindu@garvan.org.au, i.deveson@garvan.org.au

Supplementary Methods p2

Supplementary Table S1 p5

Supplementary Table S2 p6

Supplementary Figure S1 p7

Supplementary Figure S2 p8

Supplementary Figure S3 p9

SUPPLEMENTARY METHODS

Commands used for benchmark experiments on HG002 dataset

Preparation of data:

```
# merging and indexing live-converted BLOW5
slow5tools merge tmp_slow5/ -o PGXX22394_reads.blow5 -t40
slow5tools index PGXX22394_reads.blow5

# basecalling
buttery-eel -g ont-guppy-6.4.2/bin/ --config dna_r10.4.1_e8.2_400bps_hac_prom.cfg  --device 'cuda:all' -i
PGXX22394_reads.blow5 -o hg2_guppy_6.4.2_hac.fastq --qscore 9 --port 5555 --use_tcp

# mapping
minimap2 -ax map-ont -t16 --secondary=no hg38noAlt.idx hg2_guppy_6.4.2_hac.pass.fastq | samtools sort - >
hg2_guppy_6.4.2_hac_pass_minimap2.17.bam && samtools index hg2_guppy_6.4.2_hac_pass_minimap2.17.bam

# upload
aws s3 cp ${FILE} s3://gtgseq/ont-r10/NA24385/${PATH} --profile gtg-open-data
```

Fetching regions with *slow5curl*:

```
# generating the read ID list for one gene
samtools view hg2_guppy_6.4.2_hac_pass_minimap2.17.bam chr17:43,044,295-43,170,245 | cut -f 1 | sort -u >
PGXX22394_one_gene_readid.list

# generating the read ID list for hundred genes
samtools view hg2_guppy_6.4.2_hac_pass_minimap2.17.bam -L hundred_gene.random.hg38.bed -M | cut -f 1 |
sort -u > PGXX22394_hundred_gene_readid.list

# generating the read ID list for chr22
samtools view hg2_guppy_6.4.2_hac_pass_minimap2.17.bam chr22 | cut -f 1 | sort -u >
PGXX22394_reads_chr22_readid.list

# slow5curl remote index
/usr/bin/time -v ./slow5curl get https://gtgseq.s3.amazonaws.com/ont-r10-
dna/NA24385/raw/PGXX22394_reads.blow5 -t 128 --list ${READID_LIST} -o reads.blow5

# slow5curl local index
/usr/bin/time -v ./slow5curl get https://gtgseq.s3.amazonaws.com/ont-r10-
dna/NA24385/raw/PGXX22394_reads.blow5 -t 128 --list ${READID_LIST} -o reads.blow5 --index
PGXX22394_reads.blow5.idx
```

Performance scaling with number of threads when using *slow5curl*:

```
# generating the read ID list
samtools view hg2_guppy_6.4.2_hac_pass_minimap2.17.bam chr22 | cut -f 1 | sort -u >
PGXX22394_reads_chr22_readid.list

# time slow5curl
/usr/bin/time -v ./slow5curl get https://gtgseq.s3.amazonaws.com/ont-r10-
dna/NA24385/raw/PGXX22394_reads.blow5 -t ${THREADS} --list PGXX22394_reads_chr22_readid.list -o
reads.blow5 --index PGXX22394_reads.blow5.idx
```

Downloading the whole file:

```
# download file through AWS CLI
/usr/bin/time -v aws s3 --no-sign-request cp s3://gtgseq/ont-r10-dna/NA24385/raw/PGXX22394_reads.blow5 .
```

Commands used for benchmark experiments on human pangenome reference dataset

Preparation of data:

```
# FAST5 tarball conversion
tar xf ${NAME}.fast5.tar -C fast5_tmp/
slow5tools f2s fast5_tmp/ -d slow5_tmp -p40  --retain
slow5tools merge slow5_tmp/ -o ${NAME}.blow5 -t40
slow5tools index ${NAME}.blow5

# mapping
minimap2 -ax map-ont hg38noAlt.idx ${NAME}.fastq.gz -t 16 --secondary=no | samtools sort - -o ${NAME}.bam
&& samtools index ${NAME}.bam

# upload
aws s3 --profile wasabi --endpoint-url=https://s3.ap-southeast-2.wasabisys.com cp ${FILE}
s3://slow5curl/${FILE}
```

slow5curl:

```
# generating the read ID list
/usr/bin/time -v samtools view https://s3.ap-southeast-2.wasabisys.com/slow5curl/${NAME}.bam -L
hundred_gene.random.hg38.bed -M | cut -f1 | sort -u > ${NAME}_rids.list

# time slow5curl
/usr/bin/time -v slow5curl get https://s3.ap-southeast-2.wasabisys.com/slow5curl/${NAME}.blow5 -t 128 --
list ${NAME}_rids.list -o ${NAME}_reads.blow5

# time basecalling
/usr/bin/time -v buttery-eel -g ont-guppy-6.5.7/bin/ -x cuda:all --config dna_r9.4.1_450bps_sup_prom.cfg -
i "out/${NAME}_reads.blow5" -o "out/${NAME}_reads.fastq"
```

Slow5curl C library Usage

The *slow5curl* C API is built on the libraries *slow5lib* and *libcurl*. The API is split into methods for the initialisation/cleanup of: remote BLOW5 files, BLOW5 indexes, connection handles; and performing single/batch read fetches. The entire process of fetching reads is structured as so:

1. Intialise global resources
2. Initialise remote BLOW5 file
3. Download the BLOW5 index file if it is not available locally
4. Initialise the BLOW5 index
5. Initialise connection handle(s)
6. Perform single/batch fetches
7. Do something with the returned fetch
8. Cleanup initialised memory and resources

All interactions with the API should be after global resources initialised and before they are freed:

```
s5curl_global_init();
// . . . slow5curl operations
s5curl_global_cleanup();
```

Initialising resources required to fetch from a remote BLOW5 file is done simply by "opening" it from the provided URL and then "loading" its index:

```
// open remote file and load its index
```

```
s5curl_t *s5c = s5curl_open(“https://url/to/reads.blow5”);
s5curl_idx_load(s5c);
```

Fetching a single record requires a connection handle. This is exposed to give developers the flexibility of reusing connection handles and hence the ability to implement their own performance optimisations:

```
// create a connection handle for individual fetches
S5CURLCONN *conn_handle = s5curl_conn_init();

// perform a single fetch
slow5_rec_t *record = NULL;
s5curl_get(s5c, conn_handle, read_id, &record);
```

The library also offers a method to fetch reads on multiple threads. Here connection handles reside in the core data structure and records are returned in their respective data batch:

```
// initialise multiple connection handles for batch fetches
s5curl_mt_t *core = s5curl_init_mt(num_threads, s5c);

// perform a batch fetch
slow5_batch_t *db = slow5_init_batch(BATCH_CAPACITY);
s5curl_get_batch(core, db, read_ids, num_reads);
```

Both *slow5curl* and *slow5lib* share the same return data structures (*slow5_rec_t* and *slow5_batch_t*), and hence, should offer some degree of interoperability between the two libraries.

Supplementary Table S1. Data specifications.

Dataset	Platform	Pore	Sample rate	Reads	Total seq.	Read length (mean, median, max)	BLOW5 file size (zlib+svb-zd)	BLOW5 index size	BAM file size	FAST5 tarball size
~30X human genome (NA24385)	PromethION	10.4.1	4 kHz	15.3M	102.2 Gbases	6.5, 7.3, 636.6 kbases	1.1 TB	836 MB	132 GB	1.6 TB

Supplementary Table S2. Computer and connectivity specifications.

CPU (No. of cores)	RAM	OS	Ethernet connection	Computer location	Internet connection (*speedtest*)
Intel Xeon Silver 4114 CPU @ 2.20GHz (20 cores/40 threads)	384 GB	Ubuntu 18.04 LTS	Intel Ethernet Controller X710 for 10GbE SFP+	Sydney	Download: 3405.53 Mbit/s<br>Upload: 2871.61 Mbit/s<br>Ping: 1ms<br>ISP: University of New South Wales<br>Server: AARNet - Sydney

Improving ONT base-calling accuracy with software updates

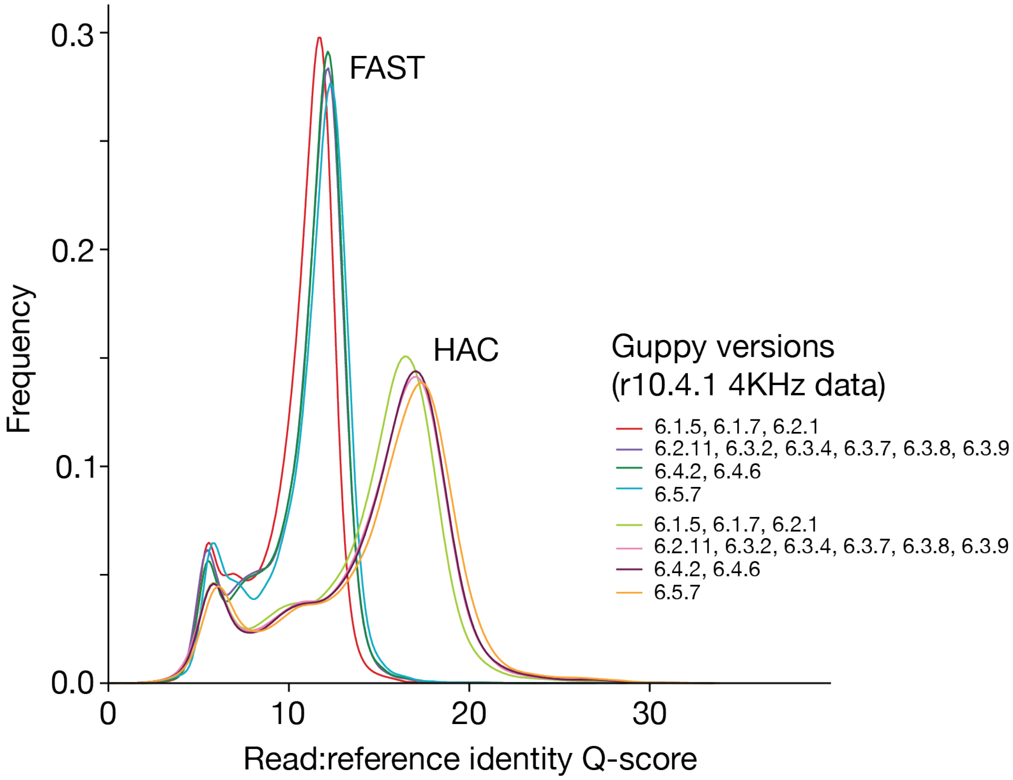

Supplementary FigS1. Assessing improvements in ONT basecalling software between Guppy versions. Frequency distributions show pre-read accuracy, as assessed by read:reference sequence identity on a Phred quality scale. Different distributions show accuracy achieved with different Guppy versions and fast vs HAC models all released within a ~1 year period (Guppy v6.2.1 (July 2022) and v6.5.7 (May 2023).

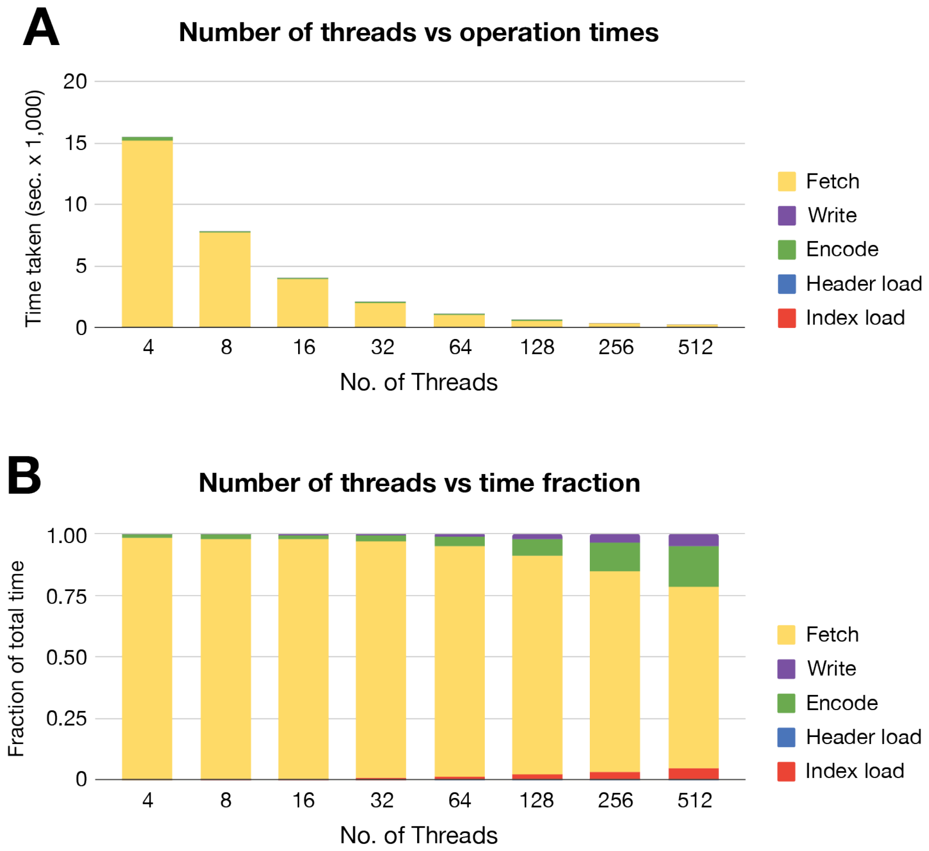

Supplementary FigS2. Evaluating the multi-threading performance in *slow5curl*. (**A**) Time taken to fetch all reads corresponding to a hypothetical gene panel comprising 100 genes from a remote whole-genome ONT sequencing file in BLOW5 format, when invoking *slow5curl* with increasing numbers of threads (*n* = 4-512). Overall times are broken down into the times taken for individual processes ('fetch', 'write', 'encode', 'header load', 'index load'). (**B**) Same as above but times for each individual process are expressed as a fraction of the total run-time, in stacked bar format.

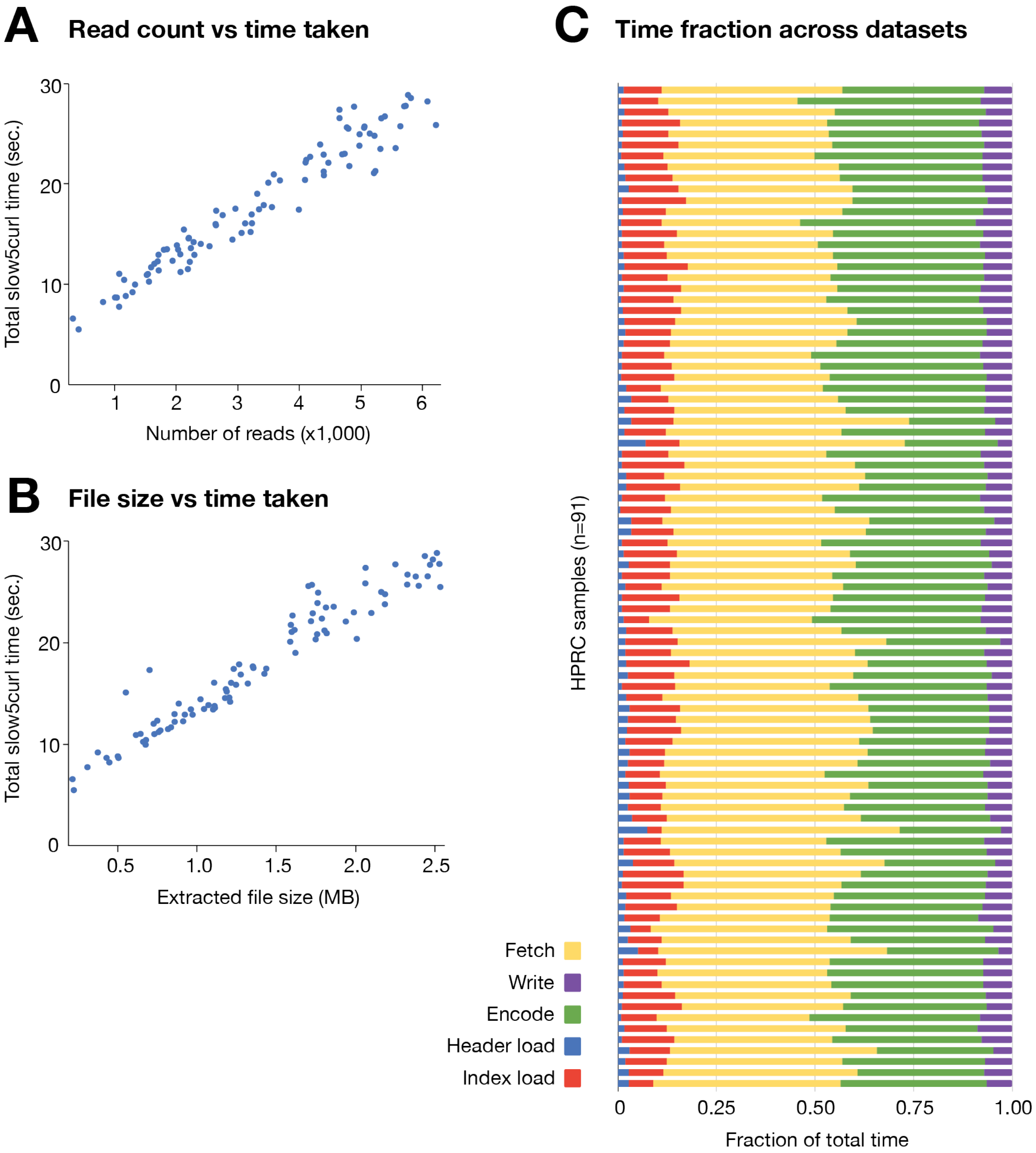

Supplementary FigS3. Evaluating *slow5curl* performance on large cohort datasets. (**A**) Time taken to fetch all signal reads corresponding to a hypothetical gene panel (n = 100 genes) from each of *n* = 91 whole-genome ONT sequencing datasets currently available via the Human Pangenome Reference Consortium (HPRC), relative to the number of signal reads being extracted for each dataset, which varies depending on the sequencing depth for each HPRC sample. (**B**) Same as above but fetch times are shown relative to extracted file sizes (in MBytes). The linear correlation observed in these two plots indicates *slow5curl* maintained stable rate of data read fetching across the full HPRC cohort. (**C**) Stacked bar chart shows the fraction of total time taken to fetch reads from each HPRC sample allocated to each individual component of the process ('fetch', 'write', 'encode', 'header load', 'index load').
