# Supplementary material for: Streamlining remote nanopore data access with slow5curl
Source: Gigascience. 2024 Apr 12;13:giae016. doi: 10.1093/gigascience/giae016 (PMC11010652; doi:10.1093/gigascience/giae016)
Supplement: giae016_Supplemental_Files [file giae016_supplemental_files.zip › SupplementaryTable3.pdf]

Genome	FAST5 tarball size (GB)	BLOW5 size (GB)	Space saving %	No. reads	Extracted BLOW5 size (GB)	Extracted No. Reads	samtools fetch time (s)	slow5curl fetch time (s)	basecall time (s)
HG00735_1	322	229	28.88	970703	1.29	2964	28.17	17.51	189.31
HG00735_3	448	314	29.91	1461840	1.65	4396	25.77	22.90	231.80
HG00735_5	335	238	28.96	1113905	1.21	3424	26.98	17.87	170.39
HG00438_1	487	341	29.98	1689007	1.64	5059	31.24	25.70	222.80
HG00438_3	450	325	27.78	1487711	1.68	4400	32.40	20.84	233.25
HG00438_5	551	388	29.58	1716121	2.08	5221	33.53	24.78	284.23
HG00621_1	576	409	28.99	1517627	2.35	4890	37.97	27.68	322.43
HG00621_3	241	174	27.80	702711	0.92	2034	23.53	13.44	129.13
HG00621_5	321	232	27.73	889955	1.22	2757	26.15	16.87	171.87
HG00673_1	437	312	28.60	1361332	1.64	4103	29.04	22.12	223.89
HG00673_3	454	325	28.41	1875969	1.52	5212	30.15	21.06	204.52
HG00673_5	351	249	29.06	1173849	1.29	3557	30.36	17.67	181.48
HG00741_1	501	363	27.54	1478970	1.97	4653	31.96	27.38	283.93
HG00741_3	577	413	28.42	1868901	2.22	5641	37.53	25.73	295.49
HG00741_5	488	344	29.51	1484047	2.00	4697	32.29	22.93	268.40
HG01071_1	395	285	27.85	1200815	1.67	3687	30.13	20.33	242.16
HG01071_3	557	394	29.26	1959255	2.15	5703	36.76	27.72	283.32
HG01071_5	504	360	28.57	1548723	1.90	4737	32.53	22.99	250.95
HG01106_1	493	347	29.61	1589757	1.72	4397	31.25	21.21	226.27
HG01106_3	624	435	30.29	1971792	2.32	5812	37.69	28.54	311.27
HG01106_5	474	334	29.54	1839676	1.54	5236	29.98	21.26	207.34
HG01123_1	323	225	30.34	1134198	1.06	3233	24.28	16.06	146.74
HG01123_3	304	211	30.59	1029606	1.16	3122	25.36	16.05	165.78
HG01123_5	303	210	30.69	838309	1.19	2648	23.83	15.86	173.66
HG01175_1	574	400	30.31	1485090	2.29	4773	36.00	25.61	320.49
HG01175_3	600	425	29.17	1760303	2.27	5332	35.61	26.52	305.25
HG01175_5	463	337	27.21	1757544	1.62	5053	30.09	25.58	216.40
HG01258_1	242	170	29.75	562241	1.05	1803	22.69	13.42	151.52
HG01258_3	228	162	28.95	548937	1.00	1853	21.94	13.47	141.68
HG01258_5	313	219	30.03	896229	1.26	2642	26.86	15.98	176.02
HG01358_1	111	76	31.53	443523	0.36	1294	17.85	9.19	53.10
HG01358_3	356	248	30.34	1066474	1.37	3347	27.44	17.45	199.19
HG01358_5	96	66	31.25	390351	0.30	1077	18.29	7.74	44.84
HG01361_1	561	393	29.95	1634811	2.06	5144	33.65	25.00	279.84
HG01361_3	518	363	29.92	2016029	1.77	5564	33.45	23.55	224.74
HG01361_5	156	109	30.13	494570	0.59	1526	19.89	10.91	80.95
HG01891_2	289	203	29.76	869405	1.06	2544	24.42	13.77	143.13
HG01891_3	460	323	29.78	1364710	1.91	4094	32.39	20.38	263.76
HG01891_4	625	435	30.40	2087994	2.37	6081	39.82	28.20	314.69
HG01928_1	92	64	30.43	237167	0.43	816	19.28	8.21	61.25
HG01928_3	244	170	30.33	875193	0.85	2402	23.70	14.00	114.61
HG01928_5	634	451	28.86	1494650	2.34	4654	36.95	26.54	317.38
HG01952_1	414	290	29.95	1444179	1.53	4177	30.38	22.68	206.87
HG01952_3	182	127	30.22	568377	0.69	1648	23.67	12.02	96.49
HG01952_5	561	406	27.63	1796893	2.22	5389	38.39	26.71	294.46
HG01978_1	253	177	30.04	756408	0.93	2298	23.73	12.91	126.97
HG01978_3	616	431	30.03	1767469	2.39	5766	39.48	28.84	318.07
HG01978_5	563	394	30.02	1608421	2.08	4976	34.99	23.79	273.14
HG02148_1	273	192	29.67	641235	1.13	2128	22.78	15.43	159.00

HG02148_3	227	158	30.40	648837	0.82	2069	20.74	12.97	117.83
HG02148_5	214	146	31.78	1186590	0.53	3064	20.50	15.10	75.70
HG02257_1	238	165	30.67	744942	0.82	2224	21.40	12.21	114.41
HG02257_3	289	200	30.80	1002236	0.98	2916	26.04	14.43	134.24
HG02257_5	224	156	30.36	1051744	0.67	2652	22.56	17.31	89.10
HG02486_1	495	342	30.91	1755832	1.68	4979	31.34	24.93	231.17
HG02486_2	198	137	30.81	646247	0.72	1944	21.40	12.32	102.29
HG02486_3	365	252	30.96	1404302	1.17	3994	27.55	17.42	165.61
HG02559_1	238	167	29.83	806965	0.78	2193	21.56	11.49	103.39
HG02559_2	233	164	29.61	796031	0.72	2073	20.97	11.20	95.83
HG02559_3	323	226	30.03	1116058	1.13	3210	25.21	15.19	154.84
HG02559_5	182	128	29.67	547598	0.63	1562	19.05	10.24	85.74
HG02572_1	191	133	30.37	546122	0.73	1719	21.49	11.36	107.38
HG02572_3	264	183	30.68	655539	1.15	2209	24.92	14.60	171.28
HG02572_5	128	89	30.47	340696	0.48	1040	18.15	8.65	69.19
HG02622_1	188	131	30.32	496827	0.80	1596	22.06	11.67	118.66
HG02622_3	217	151	30.41	535009	0.88	1716	21.73	12.92	132.17
HG02622_5	166	117	29.52	442271	0.65	1335	19.29	9.95	90.21
HG02630_1	44	31	29.55	114613	0.21	327	15.26	6.56	31.81
HG02630_3	241	169	29.88	600554	1.02	2015	22.98	13.86	151.63
HG02630_5	414	287	30.68	1392803	1.70	4114	32.13	22.38	246.89
HG02717_1	107	75	29.91	314709	0.41	1011	18.81	8.66	59.29
HG02717_3	418	289	30.86	1761238	1.52	4813	30.93	21.76	210.34
HG02717_5	486	336	30.86	1883365	1.73	5317	32.39	23.47	239.26
HG02886_1	439	306	30.30	1186817	1.73	3587	30.93	20.93	247.79
HG02886_3	485	337	30.52	1502956	1.85	4473	32.87	22.09	254.42
HG02886_5	272	193	29.04	701101	1.12	2203	26.06	14.55	153.36
HG03453_1	144	101	29.86	328190	0.62	1079	21.27	11.02	86.41
HG03453_3	220	154	30.00	509832	0.87	1703	20.87	12.26	126.58
HG03453_5	59	42	28.81	136299	0.22	420	16.07	5.48	31.08
HG03471_1	375	266	29.07	1047216	1.55	3318	29.13	19.00	229.28
HG03471_3	286	201	29.72	735128	1.15	2286	26.51	14.18	166.42
HG03471_5	379	268	29.29	1099633	1.51	3498	29.02	20.10	17.60
HG03516_1	443	308	30.47	1475078	1.68	4340	31.71	23.91	236.35
HG03516_3	553	382	30.92	2237682	1.96	6219	35.95	25.85	264.61
HG03516_5	555	386	30.45	1441728	2.41	4797	36.93	25.49	342.38
HG03540_1	346	244	29.48	1092121	1.36	3228	28.68	16.94	176.87
HG03540_3	636	448	29.56	1929425	2.41	5728	36.74	27.76	315.58
HG03540_5	120	84	30.00	365557	0.48	1185	18.45	8.81	67.80
HG03579_1	265	187	29.43	718339	1.06	2241	23.32	13.58	143.52
HG03579_3	194	135	30.41	509097	0.70	1544	21.23	11.00	98.69
HG03579_5	149	104	30.20	363158	0.65	1158	19.68	10.42	88.43
**SUM**	**31950**	**22466**		**100643467**	**120.50**	**301063**	**2486.50**	**1626.42**	**16449.68**
**MEAN**	**351.10**	**246.88**	**29.79**	**1105972**	**1.32**	**3308**	**27.32**	**17.87**	**180.77**
**MIN**	**44**	**31**	**27.21**	**114613**	**0.21**	**327**	**15.26**	**5.48**	**17.60**
**MAX**	**636**	**451**	**31.78**	**2237682**	**2.41**	**6219**	**39.82**	**28.84**	**342.38**
