# Supplementary material for: Streamlining remote nanopore data access with slow5curl
Source: Gigascience. 2024 Apr 12;13:giae016. doi: 10.1093/gigascience/giae016 (PMC11010652; doi:10.1093/gigascience/giae016)
Supplement: giae016_Response_to_Reviewer_Comments_Original_Submission [file giae016_response_to_reviewer_comments_original_submission.pdf]

Author's Response To Reviewer Comments

Please see the review response document attached as a Supplementary Material for better clarity. The following is copy pasted as requested, though not as beautifully formatted as the attached document.

Reviewer 1
1.1. The manuscript provides a detailed overview of the proposed technology, with an emphasis on reproducibility through precise software version and command line documentation. Although slow5curl is a rather simple implementation of a curl-based streaming for nanopore data, it is extensively evaluated. In this way, its value to the nanopore community is made clear.

We thank Reviewer 1 for their thorough and constructive review of our manuscript. Point-by-point responses to their comments are outlined below.

1.2. I do have a few minor comments:
Introduction: The importance of preserving raw signal data needs to be more clearly articulated. There is a view within the community that reads that have undergone high-accuracy base calling and methylation calling are sufficient for distribution and long-term storage. The clarification on the importance of raw data retention would strengthen the introduction.

At the reviewer's suggestion, we have further articulated the need to preserve raw nanopore sequencing data for future reanalysis in the Background section. This includes the addition of Supplementary FigS1 (pasted below), which assesses the impact of recent basecalling software updates on sequencing read accuracy. Please consider the following sentences that have been added:

For example, within a recent 1-year period we observed a 0.5% decrease, or 8.8% relative improvement, in the mean error rate of an identical dataset basecalled with ONT's Guppy v6.2.1 (July 2022) and v6.5.7 (May 2023; high-accuracy model; Supplementary FigS1). Rapid gains have also been made in the performance of DNA methylation detection (5mC and 5hmC), and many new tools for profiling diverse DNA and RNA modifications are released each year [3–8]. Therefore, to maximise the utility of a given dataset and to enable standardisation over time, it is important to retain ONT raw signal data for future re-analysis.

Actions: Sentences added to the Introduction emphasizing the value of retaining raw signal data. New figure assessing recent ONT basealling software improvements added (Supplementary FigS1).

1.3. Results: Please rephrase "[…] fetch a specific read(s) […]".

We have amended the sentence as follows:

The slow5curl command line tool can fetch a specific read, or group of reads, from an ONT signal dataset in binary SLOW5 (BLOW5) format[14] stored on a remote server accessible by http/https or ftp protocols (Fig1A).

Actions: Sentence amended for clarity.

1.4. Results: It should be stated more explicitly that BLOW5 is a compressed data representation and therefore suitable for streaming.

We have further articulated the suitability of BLOW5 format for streaming / efficient random access, as follows:

BLOW5 is a compressed binary format with simple file structure, which is suitable for streaming[14]. An accompanying index file describes the location of each read within the file, enabling efficient extraction of reads by random access pattern (Fig1A).

Actions: Sentence added to the Results section highlighting suitability of compressed BLOW5 for streaming.

1.5. Results: "The simple BLOW5 simple file-structure […]" -> "The simple BLOW5 file structure […]"

Thank you for picking up this typo.
Actions: Typo corrected.

1.6. Discussion: "[…] users must upload a single FAST5 tarball for a given datasets" -> "[…] users must upload a single FAST5 tarball for a given dataset"

Thank you for picking up this typo.

Actions: Typo corrected.

1.7. Discussion: While the SLOW5 ecosystem is described in detail, it would be beneficial to discuss whether there are any alternative solutions or technologies that provide a comparative perspective.

The SLOW5 data ecosystem was developed as an open-source alternative to ONT’s native data format (FAST5 / POD5) and their various associated software. The guiding philosophy and design/engineering principles are largely inspired by the seminal SAM/BAM alignment format and related tools. Therefore, these seem like the two best points of comparison to help put SLOW5 and slow5curl in context. We have added the following comparisons in the Discussion:

Slow5curl is the latest feature in the SLOW5 data ecosystem, a community-centric project designed to improve the usability of nanopore signal data (https://hasindu2008.github.io/slow5/). The initiative is inspired by the SAM/BAM alignment data format and its many associated utilities, such as the remote client feature in samtools/htslib [15], which slow5curl emulates for nanopore signal data. Efficient, remote data access by slow5tools is possible thanks to the simple SLOW5/BLOW5 file structure and accompanying index, following similar design principles to SAM/BAM. In contrast, complex file formats like ONT’s original FAST5 or new native POD5 format do not support efficient random access or indexing, thereby prohibiting efficient remote data access.

Actions: Sentences added to the Discussion comparing SLOW5 ecosystem to SAM/BAM ecosystem for comparative perspective.

1.8. Discussion: It would be interesting to discuss the possible standardization of the SLOW5 ecosystem. What is the vision? An academically centered open-source ecosystem? A proprietary system? A more "formal" standard (GA4GH, ISO/IEC)?

Thank you for this question. The SLOW5 initiative was conceived as an academically centred open-source ecosystem and we remain committed to this vision. With that said, the ideal outcome would be for SLOW5 to be adopted by ONT as a native file format used on their machines/software (instead of, or in addition to, their new ‘POD5’ format) with open source code, around which academic software could continue to be built by us and others. We have petitioned them on this, and explained the many enduring advantages of SLOW5 over FAST5/POD5, and even offered to help them with the integration. Unfortunately it seems unlikely that they will ever take us up on the offer. Although we would love to see SLOW5 adopted as a more formal standard, such as a GA4GH standard file format, it seems unlikely that this could be achieved without some endorsement/cooperation from ONT, as the major player in this space.

At the reviewer’s suggestion, we have added the following material to the Discussion on this topic:

Despite the advantages of SLOW5/BLOW5, ONT are yet to adopt the file format for direct reading/writing on their instruments or software. Therefore, we are committed to maintaining SLOW5 as a stable,

standardized, well-documented and open alternative to ONT's native data formats.

Actions: Sentences added to the Discussion outlining future vision for the SLOW5 ecosystem, from standardisation perspective.

Reviewer 2

2.1. In this manuscript, the authors demonstrate a highly streamlined method for downloading targeted subsets of raw ONT electrical signals, for re-analysis. In my view, this will be a highly useful tool for researchers working with public nanopore data, and I hope to see its widespread adoption. The benchmarks are well-described in the manuscript, and the code is publicly available and well-documented. I have no other notes or suggestions for the authors.

We thank Reviewer 2 for their thorough and constructive review of our manuscript. We are very pleased to hear that the potential value of slow5curl is clear.

Actions: No action required.

Reviewer 3

3.1. This paper introduces slow5curl, a software tool that extends slow5lib, a previous tool developed by the authors. The tool allows users to retrieve raw nanopore sequencing reads from remote BLOW5 files, a novel format that has several advantages over FAST5 and POD5, which are the most widely used formats for storing raw nanopore sequencing data. BLOW5 is not yet a standard format, but this tool could encourage its adoption and the development of similar tools in the future. The paper is well written, clear, and concise, and the tool is tested on various scenarios. The GitHub repository provides clear instructions and examples for building and using the tool.

We thank Reviewer 3 for their thorough and constructive review of our manuscript. Point-by-point responses to their comments are outlined below.

Actions: No action required.

3.2. My comments to the authors are:

Major

I am concerned about the main use case of the tool, which is to obtain a subset of raw nanopore reads that align to a specific region (e.g., a gene), in order to re-basecall them with a new software tool. This assumes that the alignment region of the original basecall is consistent with the new basecall, which may not be true. The new basecall sequences may align better to a different region, and some sequences that were not retrieved may align well to the desired region. This affects the precision and recall of the process. I would like the authors to address this issue, by either providing evidence that this is rare, or explaining why the tool is still useful despite this limitation.

We devised a new analysis to address this concern. Here we repeated the experiment presented in Fig1B using the same raw dataset basecalled with alternative basecallers and different major versions (Guppy v6.5.7 vs Dorado v7.2.13; HAC model). We recorded the list of reads that were mapped to the single-gene,

gene-panel or chr22 target regions (and hence the set of reads fetched by slow5curl) and compared these between iterations. This analysis shows that the basecalling software / version used have minimal impact, with 99.2%, 99.3% and 98.4% concordance between the two different basecallers for the single-gene, gene-panel or chr22 target regions, respectively.

We report this observation as follows:

When repeated using different basecalling software versions (Guppy v6.5.7 vs Dorado v7.2.13; HAC model), we observed high concordance in the list of reads mapped to each target region (single-gene 99.2%, gene-panel 99.3%, chr22 98.4%), meaning the basecaller version has minimal impact on the group of reads retrieved by slow5curl.

Actions: New analysis demonstrates negibile effect of re-basecalling on region-based read selection with slow5curl.

3.3. The tool depends on the availability of a BAM file for the raw reads, which is uploaded along with the BLOW5 file and its index. In the section Fetching reads from a large cohort, the authors claim that storing the raw nanopore data with its index reduces the size by 29.7% compared to FAST5. However, they do not consider the size of the BAM file, which is required for the main use case. I would like the authors to address this, by either reporting the size of the BAM files, or justifying why their size is irrelevant for this comparison.

To address this concern we have calculated the relative size of BAM, FAST5 and BLOW5 files for the NA12878 genome reference dataset used for the analysis in Fig1. The sizes are now reported in Supplementary Table S1. Notably, the BAM file is small by comparison to its corresponding FAST5 or BLOW5 dataset, and therefore there is relatively small additional cost to store BAM files alongside one’s raw data. We have added a sentence to the Results section explaining this, and reporting the observed BAM ratios for our datasets, as follows:

Basecalled reads aligned to a reference genome/transcriptome (BAM format) must also be available, stored either locally or remotely, to provide genomic coordinates for a given read. Given their small size (12.7% compared to corresponding BLOW5 or 8.9% compared to FAST5 tarball) the additional cost to do so is relatively small.

Actions: BAM and FAST5 file sizes have been added to Supplementary Table S1; additional space required to store BAM files alongside raw data is reported in the Results section.

3.4. Minor

1. In section RESULTS, in line two, delete the repeated word simple from "simple BLOW5 simple".

Thank you for picking up this typo.

Actions: Typo corrected.
