# Supplementary material for: Streamlining remote nanopore data access with slow5curl
Source: Gigascience. 2024 Apr 12;13:giae016. doi: 10.1093/gigascience/giae016 (PMC11010652; doi:10.1093/gigascience/giae016)
Supplement: giae016_Response_to_Reviewer_Comments_Revision_1 [file giae016_response_to_reviewer_comments_revision_1.pdf]

Author's Response To Reviewer Comments

Please see the attached PDF with marked up comments.
And also check our author guidelines for formatting sections:
https://academic.oup.com/gigascience/pages/technical_note
- We have done the requested markup comments

In addition, please register any new software application in the bio.tools and SciCrunch.org databases to receive RRID (Research Resource Identification Initiative ID) and biotoolsID identifiers, and include these in your manuscript. Computational workflows should be registered in workflowhub.eu and the DOIs cited in the relevant places in the manuscript. These will facilitate tracking, reproducibility and re-use of your tool.
- these have been registered and we have added them in the manuscript

Reviewer reports:
Reviewer #3: The authors correctly addressed each one of the comments I made.
From my side, no further changes are needed for publication.
Great work.
- No action required
