# Supplementary material for: Streamlining remote nanopore data access with slow5curl
Source: Gigascience. 2024 Apr 12;13:giae016. doi: 10.1093/gigascience/giae016 (PMC11010652; doi:10.1093/gigascience/giae016)
Supplement: giae016_Reviewer_1_Report_Original_Submission — Jan Voges -- 2/12/2024 Reviewed [file giae016_reviewer_1_report_original_submission.pdf]

Reviewer Report

Title: Streamlining remote nanopore data access with slow5curl

Version: Original Submission **Date:** 2/12/2024

Reviewer name: Jan Voges

Reviewer Comments to Author:

The manuscript provides a detailed overview of the proposed technology, with an emphasis on reproducibility through precise software version and command line documentation.Although slow5curl is a rather simple implementation of a curl-based streaming for nanopore data, it is extensively evaluated. In this way, its value to the nanopore community is made clear.I do have a few minor comments:Introduction: The importance of preserving raw signal data needs to be more clearly articulated. There is a view within the community that reads that have undergone high-accuracy base calling and methylation calling are sufficient for distribution and long-term storage. The clarification on the importance of raw data retention would strengthen the introduction.Results: Please rephrase "[…] fetch a specific read(s) […]".Results: It should be stated more explicitly that BLOW5 is a compressed data representation and therefore suitable for streaming.Results: "The simple BLOW5 simple file-structure […]" -> "The simple BLOW5 file structure […]"Discussion: "[…] users must upload a single FAST5 tarball for a given datasets" -> "[…] users must upload a single FAST5 tarball for a given dataset"Discussion: While the SLOW5 ecosystem is described in detail, it would be beneficial to discuss whether there are any alternative solutions or technologies that provide a comparative perspective.Discussion: It would be interesting to discuss the possible standardization of the SLOW5 ecosystem. What is the vision? An academically centered open-source ecosystem? A proprietary system? A more "formal" standard (GA4GH, ISO/IEC)?

Level of Interest

Please indicate how interesting you found the manuscript: Choose an item.

Quality of Written English

Please indicate the quality of language in the manuscript: Choose an item.

Declaration of Competing Interests

Please complete a declaration of competing interests, considering the following questions:

- Have you in the past five years received reimbursements, fees, funding, or salary from an organisation that may in any way gain or lose financially from the publication of this manuscript, either now or in the future?

- Do you hold any stocks or shares in an organisation that may in any way gain or lose financially from the publication of this manuscript, either now or in the future?
- Do you hold or are you currently applying for any patents relating to the content of the manuscript?
- Have you received reimbursements, fees, funding, or salary from an organization that holds or has applied for patents relating to the content of the manuscript?
- Do you have any other financial competing interests?
- Do you have any non-financial competing interests in relation to this paper?

If you can answer no to all of the above, write 'I declare that I have no competing interests' below. If your reply is yes to any, please give details below.

I declare that I have no competing interests.

I agree to the open peer review policy of the journal. I understand that my name will be included on my report to the authors and, if the manuscript is accepted for publication, my named report including any attachments I upload will be posted on the website along with the authors' responses. I agree for my report to be made available under an Open Access Creative Commons CC-BY license (http://creativecommons.org/licenses/by/4.0/). I understand that any comments which I do not wish to be included in my named report can be included as confidential comments to the editors, which will not be published.

Choose an item.

To further support our reviewers, we have joined with Publons, where you can gain additional credit to further highlight your hard work (see: https://publons.com/journal/530/gigascience). On publication of this paper, your review will be automatically added to Publons, you can then choose whether or not to claim your Publons credit. I understand this statement.

Yes Choose an item.
